# Mechanisms of Trifecta Valve Failure and Contemporary Approaches to Reintervention: Perceval vs Valve-in-Valve Transcatheter Aortic Valve Implantation

**DOI:** 10.1093/icvts/ivaf234

**Published:** 2026-01-07

**Authors:** Pouya Nezafati, Mohammad Azari, Sumit Yadav

**Affiliations:** Department of Cardiothoracic Surgery, Liverpool Hospital, New South Wales Health, Sydney, NSW, 2170, Australia; Department of Cardiothoracic Surgery, John Hunter Hospital, New South Wales Health, Newcastle, NSW, 2305, Australia; College of Medicine and Dentistry, James Cook University, Townsville, QLD, 4814, Australia; Department of Cardiothoracic Surgery, Liverpool Hospital, New South Wales Health, Sydney, NSW, 2170, Australia; College of Medicine and Dentistry, James Cook University, Townsville, QLD, 4814, Australia; Department of Cardiothoracic Surgery, Mater Private Hospital, Townsville, QLD 4812, Australia

**Keywords:** Trifecta valve failure, aortic valve replacement, Perceval, transcatheter aortic valve implantation

## Abstract

**Objectives:**

Aortic valve replacement with Trifecta valve provides a greater early haemodynamic performance with its externally mounted structure. However, failure rates in short-term post-implantation started to be reported, which brought great attention towards understanding the mechanisms of failure and optimal approaches for the replacement of the valve.

**Methods:**

Fifty-seven studies published in PubMed/Medline, Cochrane Central Register of Controlled Trials (CENTRAL), Embase, and ClinicalTrials.gov were explored until January 2025. Original research articles or clinical trial reports that describe Trifecta failure and studies that describe valves used to approach failed Trifecta valves were included.

**Results:**

Durability of Trifecta has shown a median range of 4 to 9 years with re-operative incidence of 7.3% to 16.9%, respectively, with smaller valves (19-21 mm) degenerating quicker. Anti-calcification compounds used and valve design are the main causes for Trifecta structural valve degeneration. Modes of Trifecta failure soon after valve implantation are leaflet(s) dehiscence or tear along the stent post and, in the longer term post-operatively, include leaflet calcification and the fibrofatty circumferential pannus development in the inflow area.

**Conclusions:**

We recommend that patients following Trifecta implantation with preoperative impaired kidneys, postoperative low effective orifice area (EOA) indexes, and prosthesis-patient mismatch be closely followed with echocardiograms to detect a possible Trifecta failure. Replacing a failed Trifecta valve with a sutureless Perceval could be an excellent option to approach a pre-existing small native annulus. Valve-in-valve transcatheter aortic valve implantation is a less favourable option due to the risk of a reduced EOA, though it may still be considered in selected patients with larger annuli.

## Graphical abstract

**Figure:**
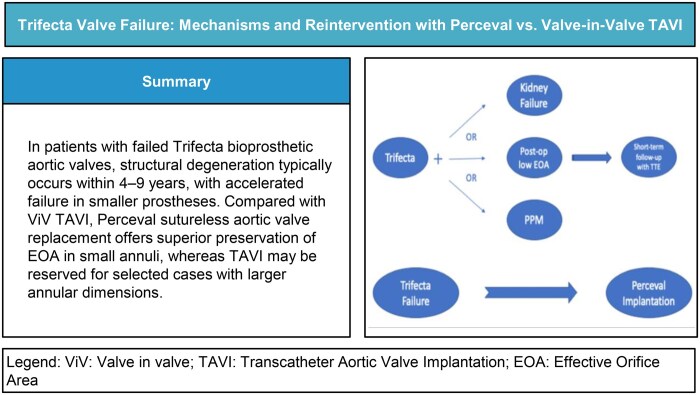

## INTRODUCTION

The growing trend for implanting biological prostheses is to inhibit long-term oral anticoagulants and to increase the quality of life.^1^ Nonetheless, biological prostheses have limited durability because of the risk of structural valve disease (SVD). Among the bioprosthetic valves, Trifecta valve (Abbott Laboratories, Illinois, United States)—made from bovine pericardial sheet on a titanium stent—has drawn great attention when initially introduced for its excellent post-implantation haemodynamic performance and recently with its concerning short-term failure rates.

## METHODS

An extensive exploration of PubMed/Medline, Cochrane Central Register of Controlled Trials (CENTRAL), Embase, and ClinicalTrials.gov was performed. Our search criteria encompassed diverse combinations and equivalents of “trifecta valve,” “trifecta valve failure,” “failed trifecta valve management,” “aortic valve replacement,” “sutureless aortic prosthesis,” “Perceval,” “bioprosthetic valves,” and “valve-in-valve TAVI.” The search period spanned from the inception of each database until January 2024.

### Study selection

We have included 57 studies that satisfied the following criteria: original research articles or clinical trial reports published in English that describe Trifecta failure and studies that describe valves used to approach failed Trifecta valves.

On the other hand, we have excluded studies that were not published in English, editorials, conference abstracts, opinion articles, case reports, animal experiments, and studies with incomplete information.

## RESULTS

### Advantages of Trifecta

The haemodynamic (low transvalvular pressure gradient) advantage of Trifecta was initially well described in different studies.^2^^,^^3^ Moreover, several studies compared the early haemodynamic performance of the externally mounted valves, like Trifecta and Mitroflow, with the internally mounted Perimount valve and showed desirable prosthetic valve-associated performance in cases with Trifecta and Mitroflow bioprostheses.^3–6^

Another study comparing Trifecta and Perimount showed significantly lower average mean peak gradient (MPG) in Trifecta than the Perimount group among all valve sizes at discharge (9.0 ± 3.1 vs 13.8 ± 4.8 mmHg, respectively; *P* < .01).^7^ They also revealed a significantly lower MPG in the Trifecta group compared to Perimount following surgery until at least 1 year after aortic valve replacement (AVR) (10.9 ± 3.8 vs 12.7 ± 5.3 mmHg, respectively; *P* < .01).^7^ Trifecta is reported to be a great option, particularly for smaller-sized annuli and in female patients, where the smaller-sized aortic valve prostheses with inner mounted leaflets can cause a high transvalvular gradient.^8^ It is suggested that the better early haemodynamics are associated with the valve design in which the valve leaflets are installed outside the sewing ring on the Trifecta valve, creating a bigger effective orifice area (EOA), as opposed to other valves in which leaflets are installed inside the sewing ring.^7^

Concern regarding the durability of the valve was initially introduced in 2014 when a 21 mm Trifecta valve was dysfunctioned 4 years later with severe stenosis.^9^ Furthermore, there have been several more reports of early, mid-, and long-term Trifecta valve failures, in which most failures occurred with smaller valve sizes.^10–23^

### Durability of Trifecta

A study on the durability of Trifecta valve has shown a 5.11% cumulative incidence of reintervention at 9 years, with a median time for reintervention of 63 months. Accordingly, smaller bioprostheses (19-21 mm) are shown to be degenerated quicker.^24^ This is supported by another research on 836 patients operated on at Southampton General Hospital^25^ and the findings of Yongue et al.^26^

Comparative studies on the durability of Trifecta with Perimount valves have shown significantly shorter durability of Trifecta. In a study by Lange et al^27^ comparing 2630 Perimount and 2423 Trifecta durability, a median of 8 years was reported in the Perimount group and 4 years in the Trifecta group. The cumulative incidence of AV reoperation was 7.3% ± 0.9% and 2.1% ± 0.3% at 5 years in the Trifecta and Perimount groups, respectively, and it was reported as a 16.9% ± 1.9% vs 3.8% ± 0.4% (*P* < .01) rate of reoperation at 8 years in the Trifecta and Perimount groups, respectively.^27^

In another study, a multicentre FinnValve registry-based study compared Trifecta and Perimount prostheses^28^ in 2216 patients with Trifecta and Perimount prostheses, reporting results comparable to those of Lange et al^27^ (reoperation rate at 7 years: 6% vs 0%, respectively).

### Cause of reintervention—SVD vs non-SVD

The cause of reintervention in cases who have a prosthetic valve prosthesis is categorized as SVD or non-SVD. The SVD presence was defined as per the guidelines^29^ as deterioration or dysfunction affecting the operating valve (except for thrombosis and infection), with deterioration of the leaflets leading to tearing, calcification, and thickening of the prosthetic valve materials, which results in haemodynamic dysfunction presenting as regurgitation or stenosis indicated by autopsy, reoperation, or clinical assessment.^10^^,^^30^

Prosthetic valve dysfunctions not due to valve tissue deterioration are considered non-SVD, including device malposition, abnormal frame expansion, prosthesis-patient mismatch (PPM), paravalvular regurgitation, and prosthetic valve endocarditis (PVE).

The possible 2 mechanisms underlying the difference in the SVD incidence between Trifecta prostheses and others are the anti-calcification compounds for leaflet preservation in the Trifecta affecting the accelerated SVD process^20^^,^^31^^,^^32^ and the design of the valve, with eliminated alignment stitches and externally mounted leaflets over the leaflets leading to premature valve failure in externally implanted bioprostheses.^23^

On the other hand, a commentary proposed that the higher redo rates may be primarily attributed to the implantation technique and the excessive pressure exerted on the strut base rather than being solely dependent on the type of bioprosthetic design.^33^ This was published in response to the FinnValve study, which highlighted a notable increase in reoperation rates among patients with Trifecta prostheses. To further investigate this, a subgroup analysis was conducted on patients who received composite grafts,^28^ as this group required no manipulation of the Trifecta prosthesis during the procedure. Even within this subgroup, there was a tendency towards higher reoperation rates among patients with Trifecta-composite grafts (mean follow-up 3.8 ± 2.1), although statistical significance was not achieved. This suggests that the implantation technique may not be the primary factor contributing to the shorter durability observed in the Trifecta bioprosthesis.^28^

### Failure modes of Trifecta

Modes of Trifecta failure soon after valve implantation are leaflet(s) dehiscence or tear along the stent post(s),^10^^,^^25^ and in the longer term post-operatively, include leaflets calcification^26^^,^^30^ and the fibrofatty circumferential pannus development in the inflow area.^20^ Failure modes are further defined as below:

#### Calcification

The predominant mechanism of SVD in Trifecta is related to calcification and is reported to be the underlying pathology for degeneration and hence reoperation after 68 months [55-77].^24^

Leaflet calcification is usually observed with prosthetic valve stenosis and in the inflow and outflow portions of the valve. Calcification happens due to a chemical interaction between the circulating calcium ions, phospholipids of the aldehyde groups, and the immunologic reaction as a result of dystrophic calcification.^32^ Despite the use of ethanol-based anti-calcification agent on the Trifecta valve, leaflet calcification still occurs,^20^ which could be due to the effect of some underlying metabolic issues, including renal impairment. Hence, patients with renal failure are at a higher risk of developing degenerated valves and should be closely followed.^32^

#### Tear

Cusp tear and dehiscence have been reported in several case reports and small series.^21^^,^^31^ In a large series of 1058 patients, sudden onset of aortic regurgitation following a partial cusp tear through a stent post was detected in 55% of the patients with Trifecta failure.^10^ This causes early reintervention of up to 4 to 5 years from implantation, with the majority of subjects presenting with severe regurgitation and more observed tears on the non-coronary cusp.^12^^,^^21^^,^^23^ The Mitroflow prostheses were found with the same mode of early failure (4.5-7 years following implantation).^34^^,^^35^ One potential mechanism of cusp tear in both valves could be explained by the similar mounting of valve leaflets externally on a titanium stent, which causes bending, tensile, and abrasion stress, especially at the cusp-free edge close to the commissure.^36^ Moreover, it might be conceivable that the initial leaflet tear of the valve prosthesis alters the haemodynamic flow dynamic, leading to a tendency for infective endocarditis and valve susceptibility to microorganisms.

#### Pannus formation

The common pathologic findings from the explanted valves are the existence of a tan-yellow, homogenous, circumferential, fibrofatty tissue attached to the inflow area of the Trifecta valve, the pannus formation. Pannus formation is known to be caused by surgical injury, gradually extending onto the sewing cuff and stent, resulting in thrombus generation^32^ and is usually observed with severe aortic regurgitation.^20^^,^^21^ Early reintervention with this mode of failure has been reported mainly in the first months after initial implantation.^35^ Patients undergoing Trifecta valve implantation can be administered with warfarin after operation for at least 3 months to inhibit early leaflet thrombosis.^32^

#### Prosthesis-patient mismatch

Average EOA for each valve size as well as the overall average size are reported to be significantly larger for the Trifecta than the Perimount group, with the reported average EOA of 0.96 ± 0.26 cm^2^ on the initial echocardiogram following the implantation of the Trifecta valve,^28^ which results in great haemodynamic valve performance. One year after operation, average EOA for all sizes of valves still showed to be significantly larger in the Trifecta compared to the Perimount group (1.64 ± 0.40 vs 1.50 ± 0.42 cm^2^, respectively; *P* = 0.01); however, EOA in the Trifecta group decreases over time thereafter.^31^

PPM is defined as an EOA that is too small in relation to the patient’s body size and cardiac output (less than 0.85).^37^ However, implantation of a recommended valve size based on industry recommendation, which takes patient body size into account, is not enough, and reports have advised other factors like end-stage renal disease (ESRD) play important roles in the EOA and hence PPM.^38^ Moreover, PPM has been reported to re-occur in cases with previous PPM following a first AVR due to the presence of underlying factors like ESRD.^39^ One strategy to overcome this issue is to consider root enlargement procedure in certain cases (eg, ESRD). On the other hand, Cleveland et al^39^ declared that Trifecta valve is highly susceptible to oversizing due to its leaflets mounted externally that cause attachment to the Valsalva sinus, which then results in incomplete leaflet coaptation and or mechanical damage during knot tying and valve insertion, which restricts leaflet motion,^39^ hence oversizing of bioprosthetic valves reduced EOAs and geometric orifice areas and enhanced pressure gradients. It is suggested that in case of a post-operative decreased EOA or PPM on echocardiogram, a close follow-up with echocardiograms is warranted to detect early failure of the Trifecta valve.

### Second-generation Trifecta

The second-generation Trifecta valve with glide technology (GT) and Linx anti-calcification (AC) technology has been introduced since 2016, with the aim to improve on the disadvantages of the first generation by providing advantages, including a higher protection against calcification and hence enhancing the longevity and durability.^23^ The Trifecta GT valve incorporates features such as a more streamlined and smaller valve holder, a screw-in handle, and a smooth delivery system, which offers the convenience of a single-cut quick-release holder, enhancing procedural efficiency.

Despite a few studies reporting on early failure of Trifecta GT with cusp tear,^11^ the reintervention rate in cases receiving the first generation of Trifecta valve is more than twice that of those receiving the Trifecta GT at the primary AVR.^40^ However, implantation of Trifecta GT valves was started later; thus, the lower rate of reintervention indicates the shorter observation length. Standardization for time may change the true reintervention rate.^11^

It is worth noting that on July 31, 2023, Abbott announced its decision to stop selling and distributing Trifecta valves, which includes first and second generations, in the United States.^41^

### Surgical and transcatheter options for the management of Trifecta valve failure

#### Surgical sutureless aortic valve—Perceval

The surgical Perceval S (LivaNova) aortic valve, as a stent-mounted, collapsible bioprosthetic valve with no sewing cuff, can be placed in a sutureless fashion using a conventional surgical technique.

The Perceval valve is rapidly deployed and does not require suturing; therefore, it can reduce the aortic cross-clamp (ACC) and cardiopulmonary bypass (CPB) times and facilitate less invasive cardiac operation.^42^ The artificial contact of bypass circuit in CPB with blood components can cause systemic inflammatory reaction, which results in diffuse microcirculatory damage and hence organ failure. Re-do operations, including cases of replacing failed Trifecta valves, are involved with some challenges, which include higher CPB and ACC time that are themselves crucial factors for mortality and morbidity after operation. The low complexity of Perceval implantation has been associated with significantly shorter CPB and ACC time and hence is a great option for the replacement of a degenerated Trifecta valve.

Using the Perceval valve allows for the implantation of prostheses with bigger diameters due to its design, leading to higher EOA, better haemodynamic performance, and smaller transvalvular gradient. This is especially very much of value in cases with a small annular size, where the indication for most initial Trifecta implantation was to overcome a possible PPM due to a known small EOA. Moreover, Perceval valve implantation can be an alternative for cases needing conventional aortic root enlargement to inhibit PPM.^43^

Successful use of the Perceval valve in failed Trifecta prostheses has been demonstrated in recent reports. Owais et al described a novel valve-in-ring implantation of Perceval into a degenerated Trifecta ring with favourable outcomes.^44^ Additionally, Kalra et al reported a cluster of early Trifecta failures, 2 of which were successfully managed with Perceval reimplantation, supporting its feasibility in complex redo cases.^45^

On the other hand, some publications report that Perceval valve implantation has been associated with a significantly higher rate of pacemaker insertions than other bioprosthetic valves.^46^ Perceval valve implantation has been associated with higher pacemaker rates than conventional surgical valves. A meta-analysis of 9492 patients reported a pooled pacemaker rate of 7% for Perceval, compared to 3%-4% for standard bioprostheses. This is likely due to radial pressure from the self-expanding stent on the conduction system.^47^

#### Non-surgical sutureless aortic valve replacement—TAVI

Transcatheter aortic valve implantation (TAVI) is a less invasive alternative to operation in cases with surgical bioprostheses failure,^48^^,^^49^ and its use is still not fully assessed in patients with failed Trifecta valves.

However, valve-in-valve (ViV) TAVI is more complicated compared to native valve TAVI and is preferably conducted by experienced teams based on the latest guidelines.^1^^,^^50^

In light of the Trifecta valve having externally mounted leaflets and a rigid sewing cuff, ViV TAVI in failed Trifecta prostheses carries specific disadvantages. These include a reduced EOA and the need for a smaller TAVI valve, especially problematic in small annuli where Trifecta valves (≤21 mm) were commonly implanted. This predisposes to PPM and suboptimal haemodynamic outcomes, with higher residual gradients linked to poorer survival. However, in patients with larger Trifecta valves (≥23 mm), ViV TAVI may still offer a reasonable alternative to reoperation, with acceptable haemodynamic performance when careful preprocedural assessment is undertaken.^48^^,^^49^

Another concerning disadvantage of ViV TAVI on Trifecta is that expanding the Trifecta leaflets mounted externally with its tall and wide stand is more susceptible to obstructing the coronary artery ostia, which could result in multiple complications,^51^^,^^52^ and hence ViV TAVI is not recommended to be conducted in externally mounted valves.

On the other hand, coronary ostial obstruction rarely occurs with ViV of the aorta over the valve of the inner-mounted Perimount valve, where the leaflets are internally mounted. Thus, the Perimount valve can be used for those who need a second valve replacement operation.^49^

In case of ViV TAVI use, the sinotubular junction, the height of the coronaries, the sinus dimension, the ascending aorta, and the valve-to-coronary (VTC) distance should be assessed to avoid complications using aortic root angiography and left anterior oblique projection with cranial angulation. The coronary complication risk is defined to be high for a VTC distance of <3 mm, intermediate at 3-6 mm, and low at >6 mm.^48^

In cases involving small Trifecta valves (≤21 mm) or narrow sinuses, Perceval AVR offers the advantage of rapid deployment and larger EOAs, and may be preferable when aortic root enlargement is necessary but a full root procedure is not feasible. Its supra-annular design facilitates annular expansion without the need for extensive reconstruction.^45^^,^^46^ However, the higher rate of permanent pacemaker implantation, often attributed to stent-related radial force on the conduction system, should be considered.^47^ ViV TAVI, while appealing for high-risk surgical candidates, presents significant challenges in failed Trifecta valves due to the external leaflet mounting and tall stent posts that increase the risk of coronary obstruction and elevated post-procedural gradients—especially in small valve sizes. ViV TAVI may be considered in Trifecta sizes ≥23 mm when preprocedural imaging confirms low coronary obstruction risk and adequate sinus dimensions.^48^^,^^49^ CT-based assessment of valve-to-coronary (VTC) distance and annular dimensions should guide procedural planning. A multidisciplinary Heart Team approach remains essential in balancing anatomical complexity with procedural risk to optimize outcomes.

## CONCLUSION

We strongly recommend that patients following Trifecta implantation with preoperative impaired kidneys, postoperative low EOA indexes, and PPM be closely followed with echocardiograms on a regular basis to detect a possible Trifecta valve failure before urgent surgery is required. Moreover, the long-term impact of the second-generation Trifecta GT should be further studied.

The Perceval valve with a rapid deployment sutureless design has a great haemodynamic profile and is an excellent option worth considering for patients at high risk for re-do Trifecta valve replacement with a high risk of PPM.

ViV TAVI is a less favourable option for failed Trifecta valves due to concerns such as coronary obstruction and PPM, particularly in patients with small annuli. However, it may be considered in selected cases with larger annuli, following evaluation by a multidisciplinary team with expertise in valvular interventions.

## Data Availability

The data underlying this article will be shared on reasonable request to the corresponding author.
